# Achievements and Current Status of the Fukushima Health Management Survey

**DOI:** 10.2188/jea.JE20210390

**Published:** 2022-12-05

**Authors:** Seiji Yasumura, Tetsuya Ohira, Tetsuo Ishikawa, Hiroki Shimura, Akira Sakai, Masaharu Maeda, Itaru Miura, Keiya Fujimori, Hitoshi Ohto, Kenji Kamiya

**Affiliations:** 1 Radiation Medical Science Center for the Fukushima Health Management Survey, Fukushima Medical University, Fukushima, Japan; 2 Department of Public Health, Fukushima Medical University School of Medicine, Fukushima, Japan; 3 Department of Epidemiology, Fukushima Medical University School of Medicine, Fukushima, Japan; 4 Department of Radiation Physics and Chemistry, Fukushima Medical University School of Medicine, Fukushima, Japan; 5 Department of Laboratory Medicine, Fukushima Medical University School of Medicine, Fukushima, Japan; 6 Department of Radiation Life Sciences, Fukushima Medical University School of Medicine, Fukushima, Japan; 7 Department of Disaster Psychiatry, Fukushima Medical University School of Medicine, Fukushima, Japan; 8 Department of Neuropsychiatry, Fukushima Medical University School of Medicine, Fukushima, Japan; 9 Department of Obstetrics and Gynecology, Fukushima Medical University School of Medicine, Fukushima, Japan; 10 Research Institute for Radiation Biology and Medicine, Hiroshima University, Hiroshima, Japan

**Keywords:** protocol, cohort study, radiation, disaster, thyroid gland

## Abstract

The Fukushima Health Management Survey (FHMS) was established in response to the Fukushima Daiichi Nuclear Power Plant accident on March 11, 2011. The primary objectives of the study are to monitor residents’ long-term health and promote their future well-being, and to determine the health effects of long-term low-dose radiation exposure. This special issue summarizes the results and current status of the FHMS and discusses the challenges and future directions of the FHMS. The FHMS, a cohort study of all people who were residents in Fukushima Prefecture at the time of the accident, consists of a Basic Survey, Thyroid Ultrasound Examination, Comprehensive Health Check, Mental Health and Lifestyle Survey, and Pregnancy and Birth Survey. The radiation exposure was estimated based on the behavioral records examined using the Basic Survey. Although the response rate was low in the Basic Survey, the representativeness of the radiation exposure data was confirmed using additional surveys. There appears to be no relationship between the radiation exposure and risk of thyroid cancer, although more thyroid cancer cases were detected than initially expected. The ongoing Comprehensive Health Check and Mental Health and Lifestyle Survey have provided evidence of worsening physical and mental health status. The Pregnancy and Birth Survey showed rates of preterm delivery, low birth weight, and congenital abnormalities similar to the national average. Considering the above evidence, the Fukushima Prefectural Government decided to end the Pregnancy and Birth Survey at the end of March 2021, as recommended by the Prefectural Oversight Committee. The framework of the FHMS has not changed, but the FHMS needs to adapt according to the survey results and the changing needs of the eligible residents and municipalities.

## INTRODUCTION

The Great East Japan Earthquake, which occurred on March 11, 2011, was followed by a tsunami that hit the Fukushima Daiichi Nuclear Power Plant and caused the release of radioactive materials. About 146,000 Fukushima Prefecture residents were evacuated from their homes by government order, and about 20,000 residents living outside the evacuation zone voluntarily evacuated due to the fear of radiation health effects.

Considering its health impact, the prefectural government decided to conduct the Fukushima Health Management Survey (FHMS) to assist with long-term management of the health of residents. The Radiation Medical Science Center for the FHMS was established at Fukushima Medical University. As described in a previous paper on the study protocol,^1^ the FHMS was designed considering the health effects of radiation following the atomic bombings of Hiroshima and Nagasaki in 1945^2^^,^^3^ and the increase in childhood thyroid cancer observed following the Chernobyl Nuclear Power Plant accident in 1986.^4^^,^^5^

The primary objectives of the FHMS were to monitor residents’ long-term health, promote their well-being, and to determine the health effects of long-term low-dose radiation exposure.^6^ A paper on the FHMS protocol was published in the *Journal of Epidemiology* in 2012.^1^ More than a decade after the radiation accident, the objectives of the FHMS have not changed, and the framework of the survey, which consists of the Basic Survey and four detailed surveys (Thyroid Ultrasound Examination, Comprehensive Health Check, Mental Health and Lifestyle Survey, and Pregnancy and Birth Survey), has been maintained. Meanwhile, 10 years after the accident, additional studies have been conducted based on the results of the respective surveys, and the direct and indirect health effects of the radiation accident are gradually becoming clearer. In addition, the awareness of the residents of Fukushima Prefecture regarding radiation effects has also gradually changed.

Therefore, in order to summarize the results of the FHMS to date, this special issue presents an overview and results of the basic survey and four detailed surveys, as well as an examination of the association between low-dose radiation exposure and the outcomes of each survey.^7^^–^^16^ Furthermore, we would like to provide an opportunity to discuss the future direction of the survey in addition to the current status and tasks of the FHMS.

## MAIN FEATURES AND PARTICIPANTS: OVERVIEW OF THE FHMS METHODS

**Figure 1.**
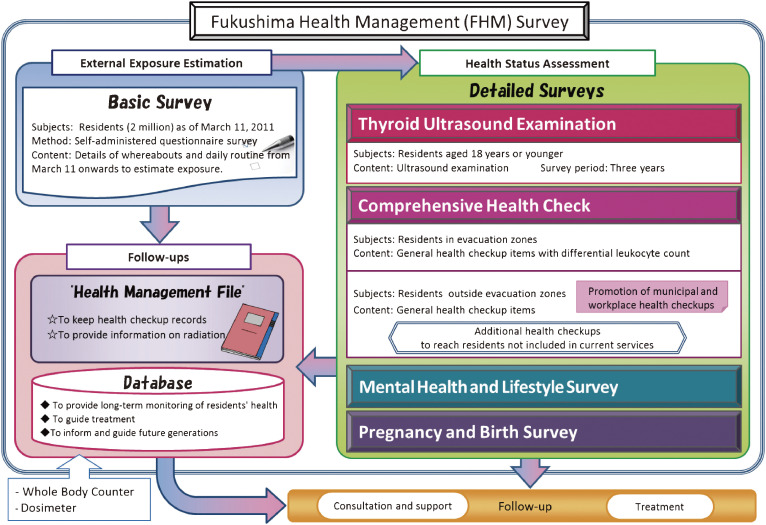
Framework of the Fukushima Health Management Survey

Protocols in the FHMS have been reported elsewhere.^1^^,^^6^ In brief, the FHMS consists of the Basic Survey^7^ and four detailed surveys: the Thyroid Ultrasound Examination,^8^ Comprehensive Health Check,^9^ Mental Health and Lifestyle Survey,^10^ and Pregnancy and Birth Survey (Figure 1).^11^^,^^12^ The eligible population of the Basic Survey was mainly individuals registered as residents in Fukushima Prefecture between March 11 and July 1, 2011 (including evacuees) and residents of other prefectures who commute to Fukushima Prefecture, totaling approximately 2.05 million people. The eligible population for the detailed survey is shown in the methods section of each survey below.

The FHMS was designed as a prospective cohort study. Epidemiologically, the exposure of interest was radiation, and the primary outcome was the incidence of certain diseases, especially cancers. Because changes of lifestyle related to evacuation of residents had the potential to affect their physical and mental health, physical and mental health conditions were included as both risk factors and outcome variables in the FHMS.

In the past 10 years, the circumstances of the evacuees have changed and have been affected by aging of the residents and the lifting of evacuation orders. Based on the results of each survey, several additional surveys have been conducted. Details of the changes in the survey in the past 10 years for each survey are provided below.

### Basic Survey

The Basic Survey estimated individual external doses of radiation in the 4 months following the 2011 nuclear power plant accident. The subjects were asked to fill out and return self-administered questionnaires on their behavioral records (records of their daily whereabouts, etc) for 4 months after the accident, and after digitizing the questionnaires, the external exposure doses for each individual were estimated using a dosimetry program developed by the National Institute of Radiological Sciences.^7^^,^^17^ It commenced on June 30, 2011. As of March 2012, the response rate was 21.9%, raising concerns about the representativeness of the whole radiation exposure in Fukushima residents. We developed a simplified questionnaire to increase the response rate.^18^ The introduction of the simplified questionnaire was the biggest change in the Basic Survey. The simplified version was only used for residents who did not move their home or workplace or moved only once within 4 months after the accident. The good validity and reliability of this new simplified version for dose estimation was confirmed.^18^ In addition, we have been conducting various activities to increase the response rate by (1) preparing a simplified version; (2) providing assistance in filling out questionnaires at various places, such as thyroid examination sites; and (3) publicizing the basic survey through mass media, such as newspapers and radio, to encourage people to submit questionnaires. Furthermore, by comparing the doses of those who had already responded to the Basic Survey with those who had not, we investigated the representativeness of the results obtained from the Basic Survey to the entire population of the Fukushima Prefecture. A total of 5,350 people were randomly selected from seven regions in the prefecture, and after checking whether or not they had already responded to the Basic Survey, a door-to-door survey was conducted to obtain answers from those who had not responded.^19^

### Thyroid Ultrasound Examination

The marked increase in thyroid cancer in children aged 4 to 5 years and older after the Chernobyl accident in 1986 raised concerns about the possibility of increased thyroid cancer prevalence in children in Fukushima. The Thyroid Ultrasound Examination has been conducted among Fukushima children aged 0–18 years.^1^ Children who lived within and outside the evacuation area whose parents were concerned about their children’s thyroid condition participated in this survey. The subjects of the baseline examination (1^st^ survey) on October 9, 2011 consisted of 367,637 residents, and the examinations were planned to be completed between 2011 and 2013, in order to check the pre-exposure status of the thyroid.^8^ Follow-up examinations (2^nd^ survey) were conducted between 2014 and 2015, a 3^rd^ survey between 2016 and 2017, and a 4^th^ survey between 2018 and 2019. In addition, survey for adults administered every 5 years was initiated in 2016.^8^

Any new findings, such as thyroid nodules, cysts, or cancers that were not found in the baseline examination, were presumed to be either induced by exposure after the baseline examination, missed in the baseline examination, or occurred as a result of natural growth in childhood.

### Comprehensive Health Check

Residents in the designated evacuation area at the time of the accident were forced to evacuate to unknown areas and experienced major life changes in their jobs, school, relationships, and their lifestyles, including diet and exercise. The evacuees required support for their physical, mental, and social health. The Comprehensive Health Check and Mental Health and Lifestyle Survey were included in the FHMS in order to improve evacuees’ physical and mental health status by reviewing their health information and assessing their lifestyle and the incidence of various diseases.^1^ The subjects were comprised of almost 210,000 individuals who were registered as residents in the evacuation area in Fukushima Prefecture between March 11, 2011 and April 1, 2012.^1^^,^^9^ This area included the 13 municipalities of Tamura City, Minamisoma City, Kawamata Town, Hirono Town, Naraha Town, Tomioka Town, Kawauchi Village, Okuma Town, Futaba Town, Namie Town, Katsurao Village, Iitate Village, and Date City. Annual health check-ups have been conducted among residents of all ages in these municipalities since 2011.

The items to be measured depended on the age of the participants, but for those aged 16 and older, the measurements included white blood cell fractions, uric acid, creatinine, and estimated glomerular filtration rate (eGFR), in addition to the usual specified health checkup measurements.^1^^,^^9^^,^^20^^,^^21^

### Mental Health and Lifestyle Survey

The Mental Health and Lifestyle Survey has been conducted annually by mail to about 210,000 residents who were living in the evacuation area at the time of the radiation accident. The eligible population of the survey is the same as the eligible population of the Comprehensive Health Check mentioned above.^1^^,^^10^

The items in the questionnaire changed slightly each year as needed, but the core questions were the same. For example, the Strength and Difficulties Questionnaire (SDQ), to assess emotional and/or behavioral problems among children, was included in the annual questionnaire for children (aged 15 years or younger), and the Kessler’s six-item questionnaire (K6), to assess depression or anxiety disorder, and the risk perception of radiation health effects were included in the annual questionnaire for those 16 years and older.^1^^,^^10^^,^^22^

In 2012, questions on the frequency of exercise were introduced for residents aged 6 years or younger, because young children were being given less opportunity to participate in outdoor exercise due to their parents’ fear of them being exposed to excess radiation, and there was concern that a lack of exercise would have an adverse effect on their mental health.^23^ Because the baseline survey in 2011 showed that those who consumed alcohol had an increased risk of serious mental illness, the CAGE questionnaire was introduced to screen participants for alcohol dependence in 2012.^24^

Furthermore, we attempted to construct a new support system that would enable us to efficiently intervene with respondents who were at risk based on the results of the K6, CAGE, SDQ, and body mass index. Specifically, we provided telephone support to respondents (almost 3,000 individuals each year) who were identified to be at risk for some kind of health problem by medical professionals and welfare personnel.^10^

### Pregnancy and Birth Survey

The eligible people of the Pregnancy and Birth Survey is pregnant women in Fukushima Prefecture and pregnant women from other prefectures who are planning to give birth in Fukushima Prefecture.^1^^,^^12^^,^^25^ The Pregnancy and Birth Survey has been conducted every year since March 11, 2011, eligible women who have registered their pregnancies during a specified period each year. Participants of the self-administered survey questionnaire were asked to respond by either mail or an online system that was available from 2016.^26^

The main items of the questionnaire were maternal factors (age, parity, and family structure), depressive symptoms, disaster-related factors, obstetric factors (medical history, type of pregnancy, illness during pregnancy, delivery method, receipt of sufficient antenatal or delivery care), and child-related factors (sex, low birth weight, and congenital anomaly).^1^

Many of the respondents to the Pregnancy and Birth Survey after the accident tended to have depressive symptoms and wrote down their serious problems in the free description section of the survey form. To support mothers with depression, we have been conducting a follow-up survey every year since 2015 for those who responded to the survey, eligible mothers in their fourth year after childbirth.^12^

Furthermore, the support system was established to alleviate anxiety by having midwives and public health nurses provide consultation and support by telephone or e-mail to respondents who needed support based on their responses in the Pregnancy and Birth Survey and the follow-up surveys.^12^

## OUTCOMES AND FOLLOW-UP: SUMMARY OF THE RESULTS OF THE FHMS

As of the end of September 2021, more than 200 papers have been published on the results of the FHMS. A summary of the results is available on the website of the Radiation Medical Science Center for the Fukushima Health Management Survey, Fukushima Medical University (http://kenko-kanri.jp/publications/). A summary of the results for each survey is shown below.

### Basic Survey

With the introduction of the simplified questionnaire, the response rate increased to 27.7% as of March 31, 2021. The external radiation doses of the respondents for the first 4 months after the accident were as follows: by the end of June 2014, 62.0%, 94.0%, and 99.4% of the respondents were exposed to <1 mSv, <2 mSv, and <3 mSv, respectively.^17^ Furthermore, the distribution was almost similar for the respondents up to the end of March 2019.^27^ The median and 90^th^ percentile values of external radiation doses during the 4-month period were 0.2 mSv to 3.8 mSv and 0.6 mSv to 7.0 mSv in municipalities in the evacuation area, while they were 0.05 mSv to 1.7 mSV and 0.1 mSv to 2.2 mSV in the other municipalities, respectively.^7^

With regard to the representativeness of individual external doses, a comparison of the doses of those who had responded to the conventional survey and those who had responded to the door-to-door survey showed that the doses of both groups were equivalent in all seven regions. Therefore, the distribution of external doses of those who have responded to the Basic Survey (approximately one quarter of all prefectural residents) might be representative of the entire population of the prefecture.

### Thyroid Ultrasound Examination

A total of 116 thyroid cancers were diagnosed in the baseline examination. As of June 30, 2020, a total of 246 examinees in the baseline, 2^nd^, 3^rd^, and 4^th^ survey at aged 25 years were determined to have nodules cytologically diagnosed as malignant or suspicious for malignancy.^8^

Regarding the association between external radiation dose assessed using the Basic Survey and thyroid cancer or suspected cancer, the analysis using the data from the baseline and 2^nd^ survey did not show any significant dose-response association between radiation dose and thyroid cancer or suspected cancer in either case.^28^^–^^31^ In the spatial analysis using data from the baseline, 2^nd^, and 3^rd^ surveys, there was no spatial clustering tendency of thyroid cancer or suspected cancer.^13^^,^^32^ Furthermore, no significant dose-response relationship was found in the analysis using dose estimate in the UNSCEAR 2013 report, which took internal exposure into account, up to the 2^nd^ survey.^33^ Therefore, the results of the analyses to date suggest that it cannot be inferred that post-accident radiation exposure is associated with the development of thyroid cancer.

### Comprehensive Health Check

Among residents aged 15 years or younger in the evacuation area, an increase was observed in the prevalence of obesity, dyslipidemia, hyperuricemia, liver dysfunction, hypertension, and diabetes mellitus after the radiation accident.^21^^,^^34^ Although the prevalence of obesity has decreased a few years after the accident, dyslipidemia has remained high.^21^^,^^34^ Furthermore, residents aged 16 years and older experienced an increase in the prevalence of obesity, dyslipidemia, hyperuricemia, liver dysfunction, hypertension, diabetes mellitus, metabolic syndrome, and kidney disease after the accident, and these increases were influenced by the evacuation.^9^^,^^35^ Between 2011 and 2017, the prevalence of people who were overweight remained the same, the prevalence of liver dysfunction decreased, and the proportion of people with controlled hypertension and dyslipidemia increased.^9^ Conversely, radiation exposure does not appear to have had any marked effect on residents’ white blood cell counts, including their neutrophil and lymphocyte counts, and lifestyle-related factors.^14^^,^^36^ Therefore, it is suggested that the increase in lifestyle-related diseases was influenced by the change in lifestyle due to evacuation. Based on the evidence of adverse effects of evacuation on health and lack of evidence for the direct effects of radiation, the Fukushima Prefectural Government has continued the Comprehensive Health Check.

### Mental Health and Lifestyle Survey

We examined the association between individual external radiation doses and psychological distress or post-traumatic stress after the accident. Although external radiation doses were not associated with psychological distress, evacuation and perception of radiation risk may increase the risk of psychological distress in women in the higher dose group.^15^

The proportion of participants aged 16 and older who may have a mood or anxiety disorder, such as depression, and were considered to need support (indicated by a K6 score of 13 or higher) was 14.6% in 2011. Since 2012, this proportion has continued to decrease and reached 7% in 2014. However, there has been no significant change in the last 5 years, and the proportion was still high compared to the national average of 3%.^10^ In particular, several mental health consequences of respondents staying outside of Fukushima Prefecture were higher than those staying inside Fukushima.^37^ In addition, it was found that those who believed that the possibility of health effects from radiation was very high were more likely to have psychological distress.^38^

The proportion of participants suspected of problem drinking (indicated by a CAGE score of 2 or higher) was highest in the year 2012 for both men and women. For men, the proportion has decreased since 2012, but for women, there has been no significant change.^10^ For both men and women, sleep problems and heavy drinking were risk factors in developing problem drinking from 2012 to 2013.^39^

On the other hand, although the proportion of people who gained more than 3 kg and lacked exercise increased after the accident up to the year 2017, the proportion of people who try to improve their lifestyle is gradually increasing, including an increase in the proportion of people who exercise regularly, a decrease in the smoking rate, and a slight improvement in sleep satisfaction.^10^

The proportion of children who were considered to be in need of support (indicated by an SDQ score of 16 or above) was high in all three age groups (4 to 6, 7 to 12, and 13 to 15 years) in 2011, especially in the age group of 4 to 6 years old (24.4%).^22^ The proportion has been declining in all age groups since 2012, but recently it has tended to be higher among school-age (7 to 15 years) children.^10^

### Pregnancy and Birth Survey

The response rate for the Pregnancy and Birth Survey was about 50.0% each year. The results of the survey from 2011 to 2018 showed that the prevalence of depressive symptoms among mothers was highest in the 2011 survey after the radiation accident and decreased over time.^12^ The 4-year follow-up data from the survey showed that the prevalence of depressive symptoms was lower than the period immediately after the childbirth and decreased over time.^12^ The proportion of mothers with radiation anxiety was higher in 2011 than in the 2014 follow-up survey,^12^ suggesting lingering effects of the radiation accident, especially among mothers who gave birth immediately after the disaster. The characteristics of mothers who received telephone childcare counseling included: first birth, cesarean section, living in an evacuation area, not being able to receive a scheduled medical checkup, and radiation anxiety.^12^

Pregnancy complications, such as gestational hypertension, respiratory diseases, and psychiatric disorders, increased in some women who were pregnant at the time of and immediately after the radiation accident.^11^ However, the direct impact on newborns, such as preterm birth, low birth weight, and congenital anomalies, was not evident in consecutive surveys, including the one immediately after the accident.^25^ Although significant differences in the incidence of preterm births and low birth weight were observed among districts, there was no significant increasing trend in the incidence of preterm births, low birth weight, and neonatal abnormalities in all six districts of Fukushima Prefecture from 2011 to 2018.^11^^,^^16^ The results of this study suggest that there is little effect of radiation accidents on the perinatal outcomes of pregnant women.

## CURRENT ISSUES AND FUTURE PROSPECTS FOR THE FHMS

### Basic Survey

As mentioned above, although the response rate in the Basic Survey was relatively low, there is no particular problem with regard to the representativeness of the respondents, and it is considered possible to examine the association with other surveys as personal data assessing external radiation doses. On the other hand, because 10 years have passed since the initial survey, memories of the respondents have become vague. Therefore, there is little need for efforts to actively increase the response rate.

Assessing radiation dose is important for examining the relationship between radiation exposure and the development of thyroid cancer, especially in children. However, about half of the children who underwent thyroid ultrasonography did not participate in the Basic Survey. There is also an opinion that thyroid-absorbed dose, rather than external exposure dose, is more necessary to evaluate the association with thyroid cancer. Therefore, an attempt has been made to evaluate internal radiation dose to the thyroid gland by combining simulation data for internal exposure and data from the Basic Survey.^40^ In the future, it may be necessary to use this evaluation method to examine the association between the results of dose assessment including internal exposure of individuals and the development of thyroid cancer.

### Thyroid Ultrasound Examination

No relationship between radiation exposure and thyroid cancer prevalence was reported in the FHMS. However, the relationship between radiation exposure and thyroid cancer should be analyzed using data of confirmatory examination in terms of the idea of “population at risk”.

In a confirmatory examination, fine-needle aspiration cytology (FNAC) was performed if needed. The number of participants who underwent FNAC decreased each year, parallel with the decreasing radiation dose level of the area. Thyroid examination was started from the relatively higher external radiation dose area to low dose one. Small number of thyroid cancer was detected due to low participation rate in low dose area. This suggested a possible relationship between radiation dose level and thyroid cancer. The participation rate was lower among older children, which may have resulted in selection bias. Furthermore, unknown confounding factors, such as lifestyle factors and participation rate of the confirmatory examination, related to thyroid examination may have contributed to the high initial thyroid cancer detection rate. The radiation doses in Fukushima were very low compared to those following the Chernobyl accident. The UNSCEAR report 2020 concluded that it is not easy to conclude whether there is a relationship between radiation exposure and thyroid cancer based on the FHMS data.^41^

The high prevalence of thyroid cancer diagnosed in the FHMS compared with cancer registration data, suggests that there may have been overdiagnosis.^42^ A simulation analysis showed that the number of observed thyroid cancer cases prevalent in the first-round examination were within the 95% confidence limits of the expected number of prevalent cases based on the cancer-progression model.^43^ The result implies that the number of observed thyroid cancer cases can be detected by the FHMS first-round thyroid examination at several sensitivities under no accident conditions. Carefully defined guidelines for the detection of thyroid cancer, especially in children and adolescents, would be necessary to avoid overdiagnosis.^43^ However, as quantifying overdiagnosis is challenging,^44^ the magnitude of overdiagnosis cannot be evaluated.

The rapid spread of COVID-19 in Japan has led to a postponement in the completion of the 4^th^ confirmatory examination. Instead of being conducted over 2 years from 2020 to 2021, it will be conducted over 3 years from 2020 to 2022. Because the possibility of overdiagnosis cannot be ruled out, we will continue to offer all eligible Fukushima residents the option of having the thyroid examination.

### Comprehensive Health Check

The most critical issue is the low participation rate of this survey in recent years. There was a decrease from 64.5% in 2011 and to 16.2% in 2019 among residents aged 15 years and younger, and a decrease from 30.9% in 2011 to 18.4% in 2019 among those aged 16 years and older. To provide appropriate support to residents, information on their health status is crucial.^6^ In order to build effective countermeasures against lifestyle-related diseases, such as diet and exercise, in each municipality, data must be representative of the eligible area. Efforts to increase the participation rate in cooperation with municipalities should be continued.

Another issue is how to prevent lifestyle-related diseases among residents in the evacuation area. The health status of the residents in the evacuation area since deterioration has not recovered even 7 years after the disaster. Thus, we will continue to provide the municipalities with information obtained from the FHMS and cooperate with their health services.

### Mental Health and Lifestyle Survey

A critical issue with the Mental Health and Lifestyle Survey is low response rate in recent years, as it decreased from 63.4% in 2011 to 15.0% in 2018 among residents aged 15 years or younger, and from 40.7% in 2011 to 19.9% in 2018 among those aged 16 years and older. Despite exhaustive efforts to increase the response rate in cooperation with municipalities, using strategies such as sending reminder postcards every year and the introduction of an online survey system in 2016, the response rate was still low. Because many items were included in the questionnaire, some residents may have found answering all the items to be burdensome. Other reasons for the low response rate are that some people believe that the accident is a fate they need to accept, although they cannot cope, or they do not want to recall accident-related issues.^6^

Under such circumstances, the 40^th^ Prefectural Oversight Committee Meeting for the FHMS, held in 2021, recommended that the Mental Health and Lifestyle Survey will be conducted on a 3-to-5-year cycle in the future.^45^ The telephone support system, which was set up by clinical psychologists and public health nurses in 2011 at the time of starting the survey will continue to provide support for as long as there is a need.

### Pregnancy and Birth Survey

The response rate was 58.2% in 2011 and continued to be almost 50% until 2019. This means that the survey and the support provided based on their responses to the questionnaire are relatively well accepted by them, and the results of the survey are relatively representative of the whole population of pregnant women.

The preterm delivery rate increased from 4.8% to 5.8%, and the rate of low birth weight increased from 8.9% to 10.1% between 2011 and 2018. The national average preterm birth rate was 5.7%, and the rate of low-birth-weight infants was 9.4%. The rate of congenital anomalies has ranged from 2.19% to 2.85% in survey participants, but the rate is similar to the 2–3% reported in the general Japanese population.^46^ Considering the above evidence, the Prefectural Oversight Committee for the FHMS recommended termination of the Pregnancy and Birth Survey at the end of March 2021.^44^ As for the follow-up survey 4 years after childbirth for support purposes, the Committee will recommend whether to continue the survey depending on the results of the 2019 and 2020 survey.

The Committee has proposed to the Fukushima Prefectural Government that further analysis comparing survey data and national data should be undertaken and that the results should be proactively disseminated in an easy-to-understand format.

### Conclusion

A decade has passed since the nuclear disaster occurred. Although the FHMS has contributed to the support of Fukushima residents by exploring their health conditions, even after 10 years, there has not been sufficient improvement regarding the physical and mental health conditions of the residents of the evacuation area, and no conclusion has been reached regarding thyroid examinations. Therefore, it is necessary to continue the survey in support of the people of Fukushima. In the next decade, the FHMS should adapt to meet the changing needs of the eligible residents and municipalities.

## References

[1] Yasumura S, Hosoya M, Yamashita S, . Study protocol for the Fukushima Health Management Survey. J Epidemiol. 2012;22:375–383. 10.2188/jea.JE20120105 22955043 PMC3798631

[2] Morgan C. Special feature. Hiroshima, Nagasaki, and the RERF. Am J Pathol. 1980;98:843–856. 6987891 PMC1903512

[3] Kodama K, Mabuchi K, Shigematsu I. A long-term cohort study of the atomic-bomb survivors. J Epidemiol. 1996;6(3 suppl):S95–S105. 10.2188/jea.6.3sup_95 8800280

[4] United Nations Scientific Committee on the Effects of Atomic Radiation. Sources and Effects of Ionizing Radiation, UNSCEAR 2008 Report (Scientific Annex D, Health Effects Due to Radiation from the Chernobyl accident). vol II. New York: United Nations; 2011.

[5] Jacob P, Bogdanova TI, Buglova E, . Thyroid cancer risk in areas of Ukraine and Belarus affected by the Chernobyl accident. Radiat Res. 2006;165:1–8. 10.1667/RR3479.1 16392956

[6] Yasumura S, Abe M. Fukushima health management survey and related issues. Asia Pac J Public Health. 2017;29:29S–35S. 10.1177/1010539516687022 28330397

[7] Ishikawa T, Yasumura S, Akahane K, . External doses available for epidemiological studies related to the Fukushima Health Management Survey: First 4-month individual doses and municipality-average doses for the first year. J Epidemiol. 2022;32(Suppl 12):S11–S22. 10.2188/jea.JE20210166 PMC9703927 36464295

[8] Shimura H, Suzuki S, Yokoya S, . A comprehensive review of the progress and evaluation of the Thyroid Ultrasound Examination program, the Fukushima Health Management Survey. J Epidemiol. 2022;32(Suppl 12):S23–S35. 10.2188/jea.JE20210271 PMC9703930 36464297

[9] Ohira T, Nakano H, Okazaki K, . Trends in lifestyle-related diseases and their risk factors after the Fukushima Daiichi Nuclear Power Plant accident: results of the comprehensive health check in the Fukushima Health Management Survey. J Epidemiol. 2022;32(Suppl 12):S36–S46. 10.2188/jea.JE20210386 PMC9703921 36464299

[10] Maeda M, Harigane M, Horikoshi N, . Long-term, community-based approach for affected people having problems with mental health and lifestyle issues after the 2011 Fukushima disaster: the Fukushima Health Management Survey. J Epidemiol. 2022;32(Suppl 12):S47–S56. 10.2188/jea.JE20210178 PMC9703932 36464300

[11] Kyozuka H, Murata T, Yasuda S, . The effects of the Great East Japan Earthquake on perinatal outcomes: results of the pregnancy and birth survey in the Fukushima Health Management Survey. J Epidemiol. 2022;32(Suppl 12):S57–S63. 10.2188/jea.JE20210444 PMC9703925 36464301

[12] Ishii K, Goto A, Yoshida-Komiya H, Ohira T, . Postpartum mental health of mothers in Fukushima: insights from the Fukushima Health Management Survey’s 8-year trends. J Epidemiol. 2022;32(Suppl 12):S64–S75. 10.2188/jea.JE20210385 PMC9703933 36464302

[13] Nakaya T, Takahashi K, Takahashi H, . Revisiting the geographical distribution of thyroid cancer incidence in Fukushima Prefecture: analysis of data from the second- and third-round Thyroid Ultrasound Examination. J Epidemiol. 2022;32(Suppl 12):S76–S83. 10.2188/jea.JE20210165 PMC9703926 36464303

[14] Sakai A, Nagao M, Nakano H, . Effects of external radiation exposure resulting from the Fukushima Daiichi Nuclear Power Plant accident on the health of residents in the evacuation zones: the Fukushima Health Management Survey. J Epidemiol. 2022;32(Suppl 12):S84–S94. 10.2188/jea.JE20210286 PMC9703929 36464304

[15] Miura I, Nagao M, Nakano H, . Associations between external radiation doses and the risk of psychological distress or posttraumatic stress after the Fukushima Daiichi Nuclear Power Plant accident: the Fukushima Health Management Survey. J Epidemiol. 2022;32(Suppl 12):S95–S103. 10.2188/jea.JE20210226 PMC9703924 36464305

[16] Yasuda S, Okazaki K, Nakano H, . Effects of external radiation exposure on perinatal outcomes in pregnant women after the Fukushima Daiichi Nuclear Power Plant accident: the Fukushima Health Management Survey. J Epidemiol. 2022;32(Suppl 12):S104–S114. 10.2188/jea.JE20210252 PMC9703922 36464294

[17] Ishikawa T, Yasumura S, Ozasa K, . The Fukushima Health Management Survey: estimation of external doses to residents in Fukushima Prefecture. Sci Rep. 2015;5:12712. 10.1038/srep12712 26239643 PMC4523853

[18] Hayashi M, Yasumura S, Kobashi G, . Dose estimation of radiation exposure for Fukushima residents after the Fukushima Dai-Ichi NPP Accident—Validity and reliability of new simplified questioner for dose estimation—. Fukushima Med J. 2015;65:149–161 (in Japanese with English abstract).

[19] Ishikawa T, Takahashi H, Yasumura S, . Representativeness of individual external doses estimated for one quarter of residents in the Fukushima Prefecture after the nuclear disaster: the Fukushima Health Management Survey. J Radiol Prot. 2017;37:584–605. 10.1088/1361-6498/aa6649 28617669

[20] Kawasaki Y, Hosoya M, Yasumura S, ; Fukushima Health Management Survey Group. The basic data for residents aged 16 years or older who received a comprehensive health check examinations in 2011–2012 as a part of the Fukushima Health Management Survey after the great East Japan earthquake. Fukushima J Med Sci. 2014;60:159–169. 10.5387/fms.2014-31 25747607

[21] Kawasaki Y, Hosoya M, Yasumura S, ; FUKUSHIMA HEALTH MANAGEMENT SURVEY GROUP. The basic data for residents aged 15 years or younger who received a comprehensive health check in 2011–2012 as a part of the Fukushima Health Management Survey after the Great East Japan Earthquake. Fukushima J Med Sci. 2015;61:101–110. 10.5387/fms.2015-13 26370684 PMC5131585

[22] Yabe H, Suzuki Y, Mashiko H, ; Mental Health Group of the Fukushima Health Management Survey. Psychological distress after the Great East Japan Earthquake and Fukushima Daiichi Nuclear Power Plant accident: results of a mental health and lifestyle survey through the Fukushima Health Management Survey in FY2011 and FY2012. Fukushima J Med Sci. 2014;60:57–67. 10.5387/fms.2014-1 25030715

[23] Itagaki S, Harigane M, Maeda M, ; Mental Health Group of the Fukushima Health Management Survey. Exercise habits are important for the mental health of children in Fukushima after the Fukushima Daiichi disaster. Asia Pac J Public Health. 2017;29(suppl):171S–181S. 10.1177/1010539516686163 28330393

[24] Ueda Y, Yabe H, Maeda M, ; Fukushima Health Management Survey Group. Drinking behavior and mental illness among evacuees in Fukushima following the Great East Japan Earthquake: the Fukushima Health Management Survey. Alcohol Clin Exp Res. 2016;40:623–630. 10.1111/acer.12984 26895603 PMC5067661

[25] Fujimori K, Kyozuka H, Yasuda S, ; Pregnancy and Birth Survey Group of the Fukushima Health Management Survey. Pregnancy and birth survey after the Great East Japan Earthquake and Fukushima Daiichi Nuclear Power Plant accident in Fukushima Prefecture. Fukushima J Med Sci. 2014;60:75–81. 10.5387/fms.2014-9 25030719

[26] Nakano H, Ishii K, Goto A, Yasumura S, Ohira T, Fujimori K. Development and implementation of an Internet survey to assess community health in the face of a health crisis: data from the Pregnancy and Birth Survey of the Fukushima Health Management Survey, 2016. Int J Environ Res Public Health. 2019;16:1946. 10.3390/ijerph16111946 31159365 PMC6603910

[27] Ishikawa T, Yasumura S, Akahane K, . The latest update on individual external doses in an early stage after the Fukushima nuclear accident. Radiat Prot Dosimetry. 2019;187:402–406. 10.1093/rpd/ncz274 31867629

[28] Ohira T, Takahashi H, Yasumura S, . Comparison of childhood thyroid cancer prevalence among 3 areas based on external radiation dose after the Fukushima Daiichi Nuclear Power Plant accident: the Fukushima health management survey. Medicine. 2016;95:e4472. 10.1097/MD.0000000000004472 27583855 PMC5008539

[29] Suzuki S, Suzuki S, Fukushima T, ; Fukushima Health Management Survey Group. Comprehensive survey results of childhood thyroid ultrasound examinations in Fukushima in the first four years after the Fukushima Daiichi Nuclear Power Plant accident. Thyroid. 2016;26:843–851. 10.1089/thy.2015.0564 27098220

[30] Ohira T, Ohtsuru A, Midorikawa S, ; Fukushima Health Management Survey group. External radiation dose, obesity, and risk of childhood thyroid cancer after the Fukushima Daiichi Nuclear Power Plant accident: the Fukushima Health Management Survey. Epidemiology. 2019;30:853–860. 10.1097/EDE.0000000000001058 31259849

[31] Takahashi H, Yasumura S, Takahashi K, . Nested matched case control study for the Japan Fukushima Health Management Survey’s first full-scale (second-round) thyroid examination. Medicine. 2020;99:e20440. 10.1097/MD.0000000000020440 32629628 PMC7337421

[32] Nakaya T, Takahashi K, Takahashi H, . Spatial analysis of the geographical distribution of thyroid cancer cases from the first-round thyroid ultrasound examination in Fukushima Prefecture. Sci Rep. 2018;8:17661. 10.1038/s41598-018-35971-7 30518765 PMC6281575

[33] Ohira T, Shimura H, Hayashi F, ; Fukushima Health Management Survey Group. Absorbed radiation doses in the thyroid as estimated by UNSCEAR and subsequent risk of childhood thyroid cancer following the Great East Japan Earthquake. J Radiat Res. 2020;61:243–248. 10.1093/jrr/rrz104 32030428 PMC7246065

[34] Kawasaki Y, Nakano H, Hosoya M, . Influence of post-disaster evacuation on childhood obesity and hyperlipidemia. Pediatr Int. 2020;62:669–676. 10.1111/ped.14162 31961051

[35] Ohira T, Nakano H, Nagai M, ; Fukushima Health Management Survey Group. Changes in cardiovascular risk factors after the great East Japan earthquake: a review of the comprehensive health check in the Fukushima health management survey. Asia Pac J Public Health. 2017;29:47S–55S. 10.1177/1010539517695436 28330394

[36] Sakai A, Ohira T, Hosoya M, ; Fukushima Health Management Survey Group. White blood cell, neutrophil, and lymphocyte counts in individuals in the evacuation zone designated by the government after the Fukushima Daiichi Nuclear Power Plant accident: the Fukushima Health Management Survey. J Epidemiol. 2015;25:80–87. 10.2188/jea.JE20140092 25311030 PMC4275442

[37] Harigane M, Takebayashi Y, Murakami M, . Higher psychological distress experienced by evacuees relocating outside Fukushima after the nuclear accident: the Fukushima health management survey. Int J Disaster Risk Reduct. 2021;52:101962. 10.1016/j.ijdrr.2020.101962

[38] Suzuki Y, Yabe H, Yasumura S, ; Mental Health Group of the Fukushima health management survey. Psychological distress and the perception of radiation risks: the Fukushima health management survey. Bull World Health Organ. 2015;93:598–605. 10.2471/BLT.14.146498 26478623 PMC4581639

[39] Ueda Y, Murakami M, Maeda M, ; Fukushima Health Management Survey Group. Maeda Risk Factors of problem drinking in the chronic phase among evacuees in Fukushima following the Great East Japan Earthquake based on a two-year cohort study: the Fukushima Health Management Survey. Tohoku J Exp Med. 2019;248:239–252. 10.1620/tjem.248.239 31406089

[40] Ohba T, Ishikawa T, Nagai H, . Reconstruction of residents’ thyroid equivalent doses from internal radionuclides after the Fukushima Daiichi nuclear power station accident. Sci Rep. 2020;10:3639. 10.1038/s41598-020-60453-0 32107431 PMC7046762

[41] UNSCEAR. Scientific. vol 2021. United Nations Scientific Committee on the Effects of Atomic Radiation; 2020:97–98 Annex B.

[42] Katanoda K, Kamo K, Tsugane S. Quantification of the increase in thyroid cancer prevalence in Fukushima after the nuclear disaster in 2011-a potential overdiagnosis? Jpn J Clin Oncol. 2016;46:284–286. 10.1093/jjco/hyv191 26755830 PMC4777612

[43] Takahashi H, Takahashi K, Shimura H, . Simulation of expected childhood and adolescent thyroid cancer cases in Japan using a cancer-progression model based on the National Cancer Registry: application to the first-round thyroid examination of the Fukushima Health Management Survey. Medicine. 2017;96(48):e8631. 10.1097/MD.0000000000008631 29310337 PMC5728738

[44] Welch HG, Black WC. Overdiagnosis in cancer. J Natl Cancer Inst. 2010;102:605–613. 10.1093/jnci/djq099 20413742

[45] Radiation Medical Science Center for the Fukushima Health Management Survey. Materials and Minutes of Prefectural Oversight Committee Meetings. 39th Report. https://www.pref.fukushima.lg.jp/uploaded/attachment/451689.pdf (in Japanese); Accessed 10/27/2021; 2021.

[46] Ishii K, Goto A, Ota M, Yasumura S, Fujimori K. Pregnancy and birth survey of the Fukushima health management survey: review of 4 surveys conducted annually after the disaster. Asia Pac J Public Health. 2017;29:56S–62S. 10.1177/1010539516684534 28330401

